# Estimating Median Survival Following Hip Fracture Among Geriatric Females: (100 – Patient Age) ÷ 4

**DOI:** 10.7759/cureus.26299

**Published:** 2022-06-24

**Authors:** Joseph Bernstein, Alexander Lee, Ianto L Xi, Jaimo Ahn

**Affiliations:** 1 Orthopaedic Surgery, Veterans Affairs Medical Center, Philadelphia, USA; 2 Orthopaedic Surgery, Perelman School of Medicine at the University of Pennsylvania, Philadelphia, USA; 3 Orthopaedic Surgery, University of Michigan, Ann Arbor, USA

**Keywords:** femoral neck fracture, hip hemi-arthroplasty, su (1): shared-decision making, post-operative mortality, total joint arthroplasties, total hip arthroplasty: tha, medical decision making, life-expectancy, hip fracture, geriatrics

## Abstract

Background:* *Estimated life expectancy for patients with geriatric hip fracture can help guide treatment selection, especially the choice between hemiarthroplasty and total hip arthroplasty for a femoral neck fracture. The purpose of the present study is to determine the survival pattern of a large cohort of geriatric female patients with hip fracture, as a function of age, for 10 years or more after injury.

Methods: All female patients between the ages of 65 and 99 who were treated surgically for hip fractures within the Veterans Affairs healthcare system from 2000 to 2010 were assessed. For every patient, the age at surgery and the age at death (if any) were recorded as of August 2021, a date at least 10.5 years after surgery.

Results: There were 818 patients in the cohort. The mean age at the time of fracture treatment was 81.2 years. Femoral neck fractures were found in 58% of the population. The survival rate for the entire group at one year was 73.7%; at two years, 62.7%; at five years, 38.6%; and at 10 years,13.7%. The median length of survival was 3.42 years, decreasing by age: for the 65-69 cohort, median survival was 8.18 years, whereas, for those 90 and above, median survival was 1.75 years. Median life expectancy could be approximated by the equation *(100 - Patient Age) ÷ 4*. Survival was not meaningfully affected by fracture type.

Conclusions:* *Geriatric hip fracture is associated with a high mortality rate. The median survival is highly correlated with age, such that an estimation equation, *(100 - Patient Age) ÷ 4, *offers a reliable shorthand for approximating it.

## Introduction

Hip fracture in elderly patients is a common condition, associated with a high mortality rate [1]. Clinicians caring for geriatric hip fractures must have reliable estimates of the patients’ associated life expectancy. This information can help guide treatment selection, especially for femoral neck fractures [2]. Displaced femoral neck fracture can be treated with a hemiarthroplasty or total hip arthroplasty, yet the long-term benefits of total hip replacements are reaped only by those who survive long-term [3,4]. Accordingly, those patients likely to live long enough to benefit from total hip arthroplasty must be identified. Nevertheless, despite the large number of studies reporting on one- and two-year survival for elderly patients with hip fracture, there is a dearth of information regarding survival beyond that [5].

The purpose of the present study is to determine survival rates, as a function of age alone, for female patients with geriatric hip fractures. Although the presence of comorbidities and other patient-specific factors will no doubt influence survival, age might provide a reasonable anchor for estimating the chances that a patient will live a fixed number of years after injury [6]. 

## Materials and methods

This study was a retrospective analysis of all female patients in the United States Veterans Affairs (VA) medical system treated surgically between January 2000 and December 2010 for hip fractures. Date of death, if any, was assessed in August 2021, representing a minimum follow-up of 10.5 years (range 10.5 - 21.3 years). The data were obtained from the VA Office of Information & Technology’s Corporate Data Warehouse (CDW). The CDW has access to clinical and administrative data pertaining to the entire VA health system, approximately 10 million patients in all. This study was deemed exempt by the local Institutional Review Board.

We collected information on all-female VA patients between 65 and 99 years of age, inclusive, identified by Current Procedural Terminology (CPT) codes for the following operations: percutaneous skeletal fixation of femoral neck fracture (27235); open treatment of femoral neck fracture (27236); treatment of peri-trochanteric femoral fracture with plate/screw-type implant (27244); and treatment of peri-trochanteric femoral fracture with intramedullary implant (27245). In addition, patients with CPT codes for hemiarthroplasty of the hip and total hip arthroplasty (27125 and 27130, respectively) were also included, provided they were associated with diagnosis-related group (DRG) codes signifying hip fracture (820.x) [7].

For every patient, age at surgery and age at death were recorded. From these two dates, the duration of survival was calculated. The patient's race, if available, was recorded. In addition, the location of the fracture was inferred from the CPT code: codes 27235, 27236, 27125, and 27130 signified femoral neck fractures, whereas 27244 and 27245 identified the peri-trochanteric fractures.

Fractional survival for the entire group was calculated for five points post-operatively: one month, one year, two years, five years, and ten years. Patients were grouped into five-year age cohorts (eg, “65 to 69”), with median and interquartile survival reported for each. Differences in survival by fracture type were assessed.

To provide a simple means of estimating median life expectancy without a need to refer to data tables directly, we fit the relationship between age and median survival for the five-year cohorts (65 to 69, 70 to 74, etc) with linear regression to generate an estimation equation. The accuracy of a simplified version of the equation, using rounded coefficients and constants, was assessed for every age bracket, 65 to 99, individually.

## Results

**Figure 1:**
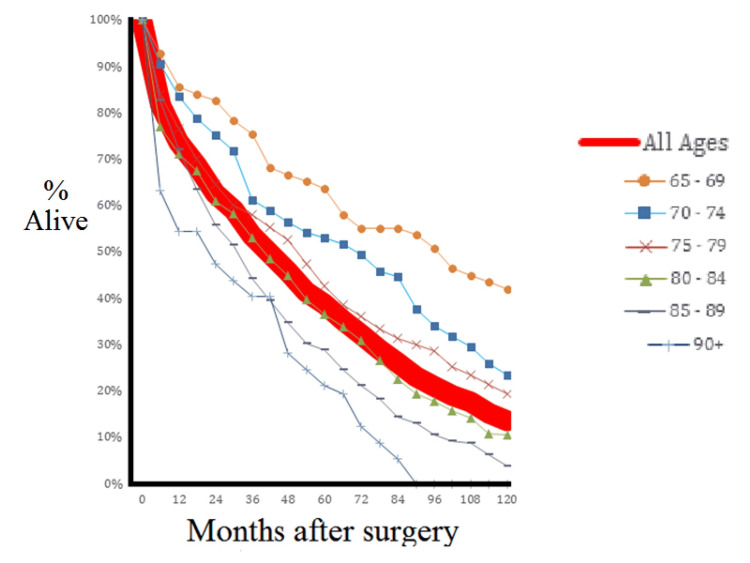
Survival after fracture

A total of 818 subjects was identified. The mean age of patients was 81.2 years (range: 65.0 to 99.2; standard deviation 6.9) and the median was 82.1 years (interquartile range 77.0 to 86.3). The rate of survival by age cohort is shown in Figure 1. 

We found a high rate of mortality in the early period after hip fracture surgery: more than 26% died within year one and 37% died by year two. Detailed survival data for the five-year cohorts is shown in Table 1. The median survival for the entire sample is 3.42 years, with median survival exceeding five years for only those patients younger than 75 years of age. Only 14% (approximately) of the entire group was alive ten years after surgery. 

**Table 1:** Survival data by five-year cohort IQR: interquartile range

Age cohort	Number of patients (%)	Median survival (with IQR)	% of the group surviving at day 30	% of the group surviving at 1 year	% of the group surviving at year 2	% of the group surviving at year 5	% of the group surviving at year 10	Number still living as of Aug 2021
All ages (years)	818 (100%)	3.42 (0.93 – 7.14)	94.13	73.72%	62.71%	38.63%	13.69%	37
65-69	69 (8.4%)	8.18 (3.09 – 13.03)	98.55%	85.51%	82.61%	63.77%	42.03%	15
70-74	85 (10.4%)	5.78 (2.10 – 9.63)	96.47%	83.53%	75.29%	52.94%	23.53%	9
75-79	150 (18.3%)	4.23 (1.19 – 8.62)	97.33%	76.67%	64.67%	42.67%	19.33%	9
80-84	249 (30.4%)	3.33 (0.77 – 6.71)	92.77%	71.08%	61.04%	36.55%	10.44%	3
85-90	208 (25.4%)	2.66 (0.87 – 5.42)	91.83%	72.12%	55.77%	28.85%	3.85%	1
90+	57 (7.0%)	1.75 (0.25 – 4.27)	91.23%	54.39%	47.37%	21.05%	0.00%	0

Among 818 subjects, 472 (58%) had femoral neck fractures and 346 (42%) fractures were peri-trochanteric. Survival for each of the different surgical procedures is shown in Tables 2, 3. The median survival for patients with femoral neck fractures, 3.8 years, was slightly higher than that of those with peri-trochanteric fractures, 3.02 years, though the former included a less invasive procedure, namely, percutaneous skeletal fixation. Median survival for patients with percutaneous skeletal fixation was 4.51 years. This duration of survival was longer than that of all other procedures except total hip replacement, whose median survival was 7.44 years.

**Table 2:** Survival for femoral neck fracture patients, by procedure CPT: Current Procedural Terminology

Procedure (CPT)	Number of patients	Fraction of cohort	Median survival, years	Interquartile range
Percutaneous skeletal fixation (27235)	78	17%	4.51	(1.73 – 8.08)
Open treatment of femoral neck fracture (27236)	251	53%	3.58	(0.76 – 6.45)
Hemiarthroplasty of the hip (27125)	113	24%	3.66	(1.15 – 8.05)
Total hip arthroplasty (27130)	30	6%	7.44	(1.69 – 11.17)
All femoral neck fracture procedures excluding percutaneous skeletal fixation (27235)	394	83%	3.66	(0.91 – 7.43)
All femoral neck fracture procedures combined	472	100%	3.8	(0.94 – 7.52)

**Table 3:** Survival for peri-trochanteric fracture patients, by procedure

Procedure	Number of patients	Fraction of cohort	Median survival, years	Interquartile range
Treatment of peri-trochanteric femoral fracture with plate/screw type implant (27244)	220	64%	3.06	(1.01 – 6.81)
Treatment of peri-trochanteric femoral fracture with intramedullary implant (27245)	126	36%	2.77	(0.73 – 6.44)
Both peri-trochanteric fracture codes combined	346	100%	3.02	(0.90 – 6.61)

The best-fit regression line for median survival was calculated to be -0.505 + 0.2417 (100 - age), with an R2 of 0.94 with a standard error of 1.3 years. A simplified version of this equation, *Life Expectancy = (100 - Patient Age) ÷ 4,* was accurate to within two years of the observed median survival for 20 of the 25 ages, 65 to 89, and for all ages 75 to 89.

Because there were 215 patients for which race was unknown (22%), analysis based on race was not conducted.

## Discussion

In the case of displaced fractures of the femoral neck in geriatric patients, the selection of treatment can be difficult. Surgeons may select the easier and cheaper option of hemiarthroplasty, especially if the patient does not have high functional demands. They may also select the more expensive and difficult to perform option of total hip arthroplasty, but surgeons should reserve that option only for patients likely to survive long enough to reap its benefits. As such, the process of selecting a treatment must consider anticipated survival.

In this study, we not only confirmed that there is a high rate of mortality in the first two years after hip fracture surgery [8,9] but also that there is a high rate of mortality in the first month too. This is valuable information for medical decision making as patients facing a high risk of imminent death might be better treated with palliative care and no surgery [10,11]. 

At the other extreme, patients with longer life-expectancies might be better candidates for total hip arthroplasty, and not hemiarthroplasty. That's because total hip arthroplasty, though initially more complicated and costly than hemiarthroplasty, offers better long-term function (as seen in higher Harris Hip Scores) and a lower rate of reoperations [3,4]. And, simply put, if the advantages of an operation are reaped only in the long run, that procedure is more indicated for patients apt to live long enough to benefit from it. Accordingly, predicting likely survival after hip fracture surgery is paramount.

In this study, we describe the 10-year survival patterns of a group of 818 female patients with operative treatment of hip fracture. The rates of survival by five-year cohort are reported explicitly here, but clinicians at the bedside can generate a reasonable approximation of median life expectancy by using the equation, *Life Expectancy = (100 - Patient Age) ÷ 4*. 

We confirmed a high rate of mortality for all patients of all ages. On the other hand, 13% of all patients and 42% of those aged 65-69, lived a decade or more after injury, confirming as well that some patients will live long enough to benefit from total hip replacement.

To our knowledge, based on investigation, there is only one prior study reporting long-term survival by age-cohorts in a large sample of female patients [8]. In that study, slightly longer survival times were seen (eg, 79% at one year and 48% at five years) but 9% their cohort (66 of 766 patients) were younger than age 65, skewing the observed survival. Indeed, 30 of those 66 relatively young patients were still alive 22 years or more after injury.

Limitations

There are limitations of this study. First, this was an investigation restricted to VA patients. VA patients are known to have “large differences in sociodemographic status [and] health status” as compared to general patient population [12,13]. As such, extrapolation of VA findings to the general population may be limited [14]. 

This study is also limited by its simple, indeed primitive, methods of analysis: age was the only variable considered. There were no attempt to measure the effect of clinical variables and comorbidities on life expectancy. Although age is perhaps the best starting point for predicting life-expectancy, it is only a start. Also, use of median values are best limited to anchoring an initial estimate, one to be refined with additional patient specific information. (By definition, half of the group lives more than the median and half lives less. As such, the median is highly likely to not match precisely the survival of any specific individual. Rather, the median identifies a starting point estimate that can be perfected with further clinical knowledge.) 

Last, this study reported only operative cases, whereas it is likely that sicker patients are treated without surgery. Accordingly, the true rates of survival for all geriatric hip fracture patients are lower than what is reported here.

## Conclusions

For much of medical history, when treatments were less effective, issuing a prognosis was the primary value added by physicians. These days, predicting life expectancy is still important, especially for an elderly patient presenting with a hip fracture. That is because total hip arthroplasty (one of the possible treatments for this injury) offers the particular benefit of prosthetic longevity to only those patients who survive long enough to reap it. Such patients must therefore be detected. (Note that the number of years that qualify as "long enough" is based on an expected utility analysis that is beyond the scope of this report.)

In this study, although many elderly patients with hip fractures were seen to have not survived a full year, a significant fraction of the group lived more than a decade following injury. The patients with the greatest potential for long-term survival must be identified, so they may be considered for total hip replacement. The results here suggest that patients’ median survival prognosis can be approximated by the equation *Life Expectancy ≈ (100 - Patient Age) ÷ 4*. Additional analysis can provide the necessary refinements from that base.
